# Contribution of Large Region Joint Associations to Complex Traits Genetics

**DOI:** 10.1371/journal.pgen.1005103

**Published:** 2015-04-09

**Authors:** Guillaume Paré, Senay Asma, Wei Q. Deng

**Affiliations:** 1 Department of Pathology and Molecular Medicine, McMaster University, Hamilton, Canada; 2 Population Genomics Program, Department of Clinical Epidemiology and Biostatistics, McMaster University, Hamilton, Canada; 3 Population Health Research Institute, Hamilton Health Sciences and McMaster University, Hamilton, Canada; 4 Thrombosis and Atherosclerosis Research Institute, Hamilton, Canada; 5 Department of Statistical Sciences, University of Toronto, Toronto, Canada; National Human Genome Research Institute, UNITED STATES

## Abstract

A polygenic model of inheritance, whereby hundreds or thousands of weakly associated variants contribute to a trait’s heritability, has been proposed to underlie the genetic architecture of complex traits. However, relatively few genetic variants have been positively identified so far and they collectively explain only a small fraction of the predicted heritability. We hypothesized that joint association of multiple weakly associated variants over large chromosomal regions contributes to complex traits variance. Confirmation of such regional associations can help identify new loci and lead to a better understanding of known ones. To test this hypothesis, we first characterized the ability of commonly used genetic association models to identify large region joint associations. Through theoretical derivation and simulation, we showed that multivariate linear models where multiple SNPs are included as independent predictors have the most favorable association profile. Based on these results, we tested for large region association with height in 3,740 European participants from the Health and Retirement Study (HRS) study. Adjusting for SNPs with known association with height, we demonstrated clustering of weak associations (*p* = 2x10^-4^) in regions extending up to 433.0 Kb from known height loci. The contribution of regional associations to phenotypic variance was estimated at 0.172 (95% CI 0.063-0.279; *p* < 0.001), which compared favorably to 0.129 explained by known height variants. Conversely, we showed that suggestively associated regions are enriched for known height loci. To extend our findings to other traits, we also tested BMI, HDLc and CRP for large region associations, with consistent results for CRP. Our results demonstrate the presence of large region joint associations and suggest these can be used to pinpoint weakly associated SNPs.

## Author Summary

It is widely accepted that genetics influences a broad range of human traits and diseases, yet only a few genetic variants are known to determine these traits and their impact is modest. In this report, we made the hypothesis that combining information from a large number of genetic variants would help better explain how they together contribute to traits such as height. To do so, we first had to select a proper method to integrate large numbers of genetic variants in a single test, here named “large region joint association”. Next, we tested our method on height in 3,740 European participants from the Health and Retirement Study. We showed that the contribution of regional associations to variation in height was 17.2%, as compared to the 12.9% explained by known genetic determinants of height. In other words, the joint effect of multiple genetic variants integrated together contributed to a substantial fraction of the genetics of height. These results are significant because they can help identify new genes or genetic regions associated with human traits or diseases. Conversely, these results can be used to better understand genes that we already know are associated. Furthermore, our results provide insights on how traits are genetically determined.

## Introduction

It is widely accepted [1,2] that a large fraction of the variance of complex traits is explained by common genetic variants, yet a relatively small number have been associated at genome-wide significance and they collectively explain only a minor fraction of the total predicted heritability. The discrepancy between predicted heritability from population studies and variance explained by known genetic determinants has been termed the “missing heritability”, and is currently one of the most pressing issues in human genetics [3]. Among others, it has been proposed that weak, yet undetected, associations underlie complex trait heritability [2], and that interaction of multiple genetic variants could potentially account for some of the missing heritability [4]. Clustering of weak associations within defined chromosomal regions has been suggested [5] and indeed, SNPs at known GWAS loci have been shown by variance component approaches to contribute significantly to heritability [6]. Furthermore, conditioning on known genetic determinants can reveal novel associations [7], coding and *cis*-regulatory variants have been shown to modify the functional effect of each other [8], and genetic variants can impact *cis* gene expression over regions spanning hundreds of kilobases [9,10]. Nonetheless, while variance component methods can estimate overall variance explained by genetic variants based on genetic similarity between individuals, no method has explored the individual and aggregate contribution of SNPs to large region associations. We hypothesized that joint association of multiple weakly associated variants over large chromosomal regions contributes to complex traits variance. Such joint associations will be best characterized by association models that are robust to linkage disequilibrium (LD) structure and the presence of gene-gene interactions.

Many regional association tests have been proposed [6,11]. However, no report has systematically evaluated the power of commonly used statistical models to capture the phenotypic variance explained by SNPs over large regions, while taking into account both the diploid nature of our genome and the possibility of long-range *cis*-interactions. In fact, most association studies have analyzed SNPs individually either with or without follow-up conditional analyses [7]. Tregouët *et al*. [12] was the first to report haplotype testing on a genome-wide basis, but the proposed method assumed short haplotypes, and as such did not test for aggregations of weak association signals over extended regions nor long-range *cis*-interactions. A recent report [13] described a method for multi-SNP association where SNPs are first pruned to meet a minimum *p*-value threshold in univariate analysis and to ensure linkage disequilibrium *r* ^2^<0.1. However, methods to capture genetic variance explained by multiple variants clustering in extended chromosomal regions when no single variant is strongly or modestly associated by itself have not been fully explored. There is thus a need for methods that leverage the potential aggregation of functional variants within extended genetic regions, irrespective of linkage disequilibrium or whether these variants contribute to phenotypic variance independently or through *cis*-interactions.

In this report, we first characterized the ability of commonly used genetic association models to capture the variance explained by large region joint associations. Through theoretical derivation and simulations, we showed that multivariate linear models where multiple SNPs are included as independent predictors have the most favorable profile under a variety of association scenarios. Furthermore, we showed that multivariate linear models are equivalent to variance component models when the SNPs tested are in complete linkage equilibrium. Informed by these results, we tested for regional association with height in 3,740 European participants of the Health and Retirement Study (http://hrsonline.isr.umich.edu/). Height was chosen because of its high heritability, demonstrated polygenic genetic architecture [2,14], and the presence of 180 known association loci [15]. We confirmed clustering of weak associations near known height loci, demonstrated that large region joint associations can explain a large fraction of phenotypic variance, and showed that suggestively associated regions are enriched for known height loci. To extend our findings to other traits, we also tested Body Mass Index (BMI), High-Density Lipoprotein cholesterol (HDLc) and C-reactive Protein (CRP) for large region associations.

## Results

### Notations and Background

Let matrix ***H***_(k×m)_ represent all possible haplotypes defined by *m* biallelic SNPs, where *k* = 2^m^ is the number of possible haplotypes. The reference and alternate alleles of a SNP are coded as 0 and 1, respectively. The corresponding population haplotype frequencies are given by a vector of length *k*:

$$\pi =[\pi_{1},\pi_{2},\ldots ,\pi_{k}].$$

Further, for *k* possible haplotypes, we define the matrix ***D*_(*n×k*)_** to represent the diplotypes, i.e. the combinations of two haplotypes for each of the *n* individuals. The row entries of matrix ***D*** correspond to the presence or absence of a particular haplotype and are indicated by possible values of 0, 1, or 2, such that the sum of each row is 2. In other words, if the diplotype of the *i* ^th^ individual is composed of a pair of two distinct haplotypes corresponding to the *u* ^th^ and *v* ^th^ columns of matrix ***H***, then the entries *D*_*iu*_ and *D*_*iv*_ take the value 1. On the other hand, if the individual is homozygote for the *u* ^th^ haplotype then we have *D*_*iu*_ = 2 Absence of all other haplotype is indicated by 0. In addition, the unphased genotype matrix ***G*** is given by:

$$G_{\left(n\times m\right)}=DH=\left[\begin{array}{l} g_{11}\;g_{12}\;\cdots \;g_{1m}\\ g_{21}\;g_{22}\;\cdots \;g_{2m}\\ \;\;\;\vdots \;\;\;\;\;\;\vdots \;\;\;\;\;\;\vdots \;\;\;\;\;\vdots \\ g_{n1}\;g_{n2}\;\cdots \;g_{nm} \end{array}\right]$$

where rows represent the number of alternative alleles at each of the *m* SNPs for a given individual.

### Genetic Association Models

We herein refer to the true underlying genetic association model **Y = *Dβ + ε*** as the haplotype model, where ***D*** is the previously defined matrix of true (unobserved) haplotypes and ***β*** a vector of unknown haplotype effects. Multiple linear regression is frequently used in genetic association studies to model or test the presence of genetic effects. These models assume that a linear relationship exists between some phenotype ***Y*** and the observed genetic covariates ***X***, which can be genotypes or inferred haplotypes. Let’s posit the following linear regression model

Y = XB + ε,

where we assume the trait ***Y*** is standardized to have mean 0, variance 1 and **ε** is the standard normally distributed random error. The unknown coefficients (vector) ***B*** represent the real genetic effects and the maximum likelihood estimate can be found by $\hat{B}\;=\;{\left(X'X\right)}^{-1}X'Y$. The phenotypic variance explained by the real genetic effects then takes the form $\sigma_{X}^{2}\;=\;B'X'XB$. This general model can be adapted to specific genetic association models by varying the definition of ***X***, in this manuscript defined as additive and interaction effect models, genotypic model, and haplotype probability model. Briefly, in the additive model ***X*** = ***G*** such that it is equivalent to a multivariate linear model with the number of alternative allele at each SNP as independent variables and overall statistical significance determined with an *F*-test. The interaction model combines the additive model and all pairwise SNP-SNP interactions. The genotypic model considers all possible genotypes as categorical variables. The haplotype probability model uses the probability of each haplotype pair from unphased genotype data as independent variables. Finally, the variance component model estimates genetic variance explained using pairwise genetic similarity between individuals. A detailed description of models is provided in Methods.

### Measure of Non-additivity

We consider a trait to follow a “strictly additive” model if it can be appropriately described by a linear combination of the number of alternative alleles at each SNP (i.e. when no SNP x SNP interaction, dominant, recessive or haplotype effects are present). It is relevant to investigate conditions where the strictly additive model does not adequately explain phenotypic variation. Deviation from the additive model, or “non-additive effects”, could indicate the presence of either non-linear (i.e. recessive or dominant) or interaction effects. We herein define a measure of non-additivity $\tau^{'}\;=\;\frac{\tau }{max\left(\tau \right)}$ where $\tau \;=\;\frac{\sigma_{H}^{2}-\sigma_{G}^{2}}{\sigma_{H}^{2}}$, $\sigma_{H}^{2}$ is the variance explained by underlying haplotypes, and $\sigma_{G}^{2}$ the variance explained by genotypes using an additive genetic association model. Therefore, *τ′* will be equal to 0 when a strictly additive model completely captures the phenotypic variance explained by underlying (unobserved) haplotypes and *τ′* will be equal to 1 when deviation from a strictly additive model is maximized (since multivariate linear models always capture at least a minimal fraction of underlying genetic variance).

### Regional Association Involving Common Genetic Variants

We first tested the ability of genetic association models to estimate regional genetic effects under plausible scenarios involving common variants (minor allele frequency>0.01). We assumed a quantitative trait to be genetically determined according to the underlying (unobserved) haplotype model **Y = Dβ + ε** where 2 SNPs define 4 possible haplotypes. To compare these models on equal footing, we fixed the proportion of variance explained by haplotypes at 0.006 and varied haplotype effects such that the non-additivity parameter(τ′) ranged from 0 to 1 (S1 Fig). We then added “nuisance” SNPs (i.e. not associated with the trait), ensuring the pairwise LD between all pairs of SNPs was either *r* ^2^ = 0 or 0.2. Finally, we calculated power for a sample of 5,000 individuals while arbitrarily setting the *p*-value threshold at 5x10^-5^, corresponding to a suggestive regional association.

**Fig 1:**
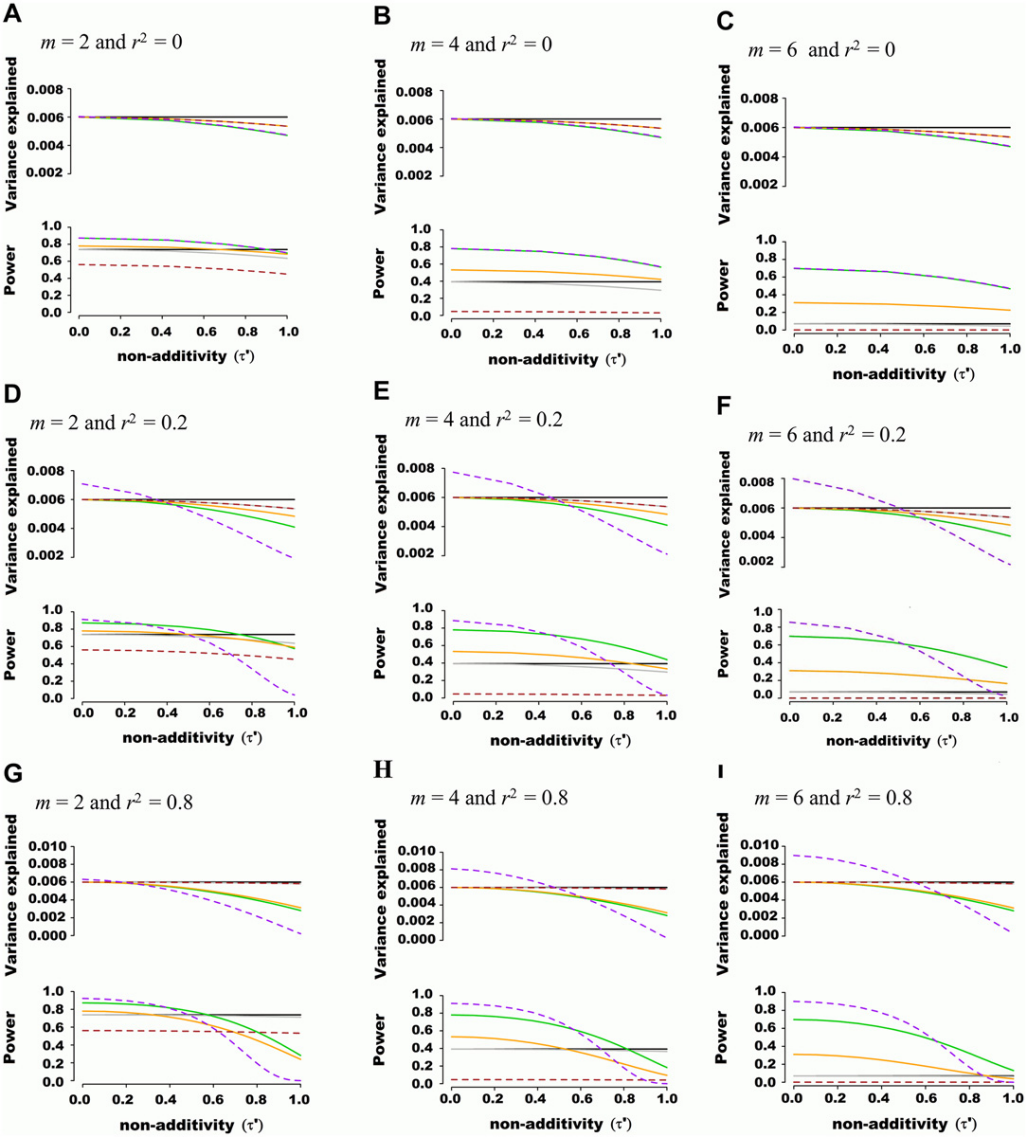
Estimated variance explained and power as a function of non-additivity measure τ*´*. A quantitative trait was assumed to be genetically determined according to the underlying (unobserved) haplotype model ***Y*** *=* ***Dβ*** *+* ***ε***, where 2 SNPs define 4 possible haplotypes. The proportion of variance explained by haplotypes was fixed at 0.006 while haplotype effects varied such that the non-additivity parameter (τ*´*) ranged from 0 to 1. Two non-associated nuisance SNPs were added in (B), (E) and (H), bringing the total number of SNPs to *m* = 4, and four non-associated nuisance SNPs were added in (C), (F) and (I), bringing the total number of SNPs to *m* = 6. In (A), (B) and (C), the frequency of haplotypes was fixed such that pairwise SNP linkage disequilibrium *r* ^*2*^ = 0, in (D), (E) and (F) frequencies were fixed such that *r* ^*2*^ = 0.2, and in (G), (H) and (I) frequencies were fixed such that *r* ^*2*^ = 0.8. Each line corresponds to a genetic association model, with the underlying haplotype model in black, the additive model in green, the interaction effects model in orange, the genotypic model in dashed brown, the haplotype probability model in grey, and the variance-component model in dashed purple. The upper panel of each figure illustrates the estimated proportion of phenotypic variance explained by joint association as a function of non-additivity τ*´*. The lower panel illustrates the power to detect such joint association with a *p*-value threshold of 5x10^-5^.

The additive model (i.e. where the number of minor alleles at each SNP is included as independent predictors in a multivariate linear model) showed a favorable balance of power and unbiasedness of genetic variance estimates. For instance, the estimated genetic variance was similar to the variance explained by underlying haplotypes (i.e. 0.006) when non-additivity was modest (τ′<0.4), irrespective of the number of nuisance SNPs or LD structure (representative example illustrated in Fig 1). The haplotype probability and interaction (i.e. additive model plus all pairwise interactions) models provided accurate estimates of genetic variance but had inferior power, especially when nuisance SNPs were added. The genotypic model also accurately estimated genetic variance, but had the lowest power among methods tested. This was due to the high number of degrees of freedom involved, which also explained the lower power of the haplotype and interaction models when nuisance SNPs were added. As predicted, the variance component model behaved identically to the additive model when SNPs were in linkage equilibrium. However, when LD was present, variance component models tended to either under or overestimate genetic variance. No type I error inflation was observed under the null hypothesis of no association, irrespective of linkage disequilibrium.

**Fig 2:**
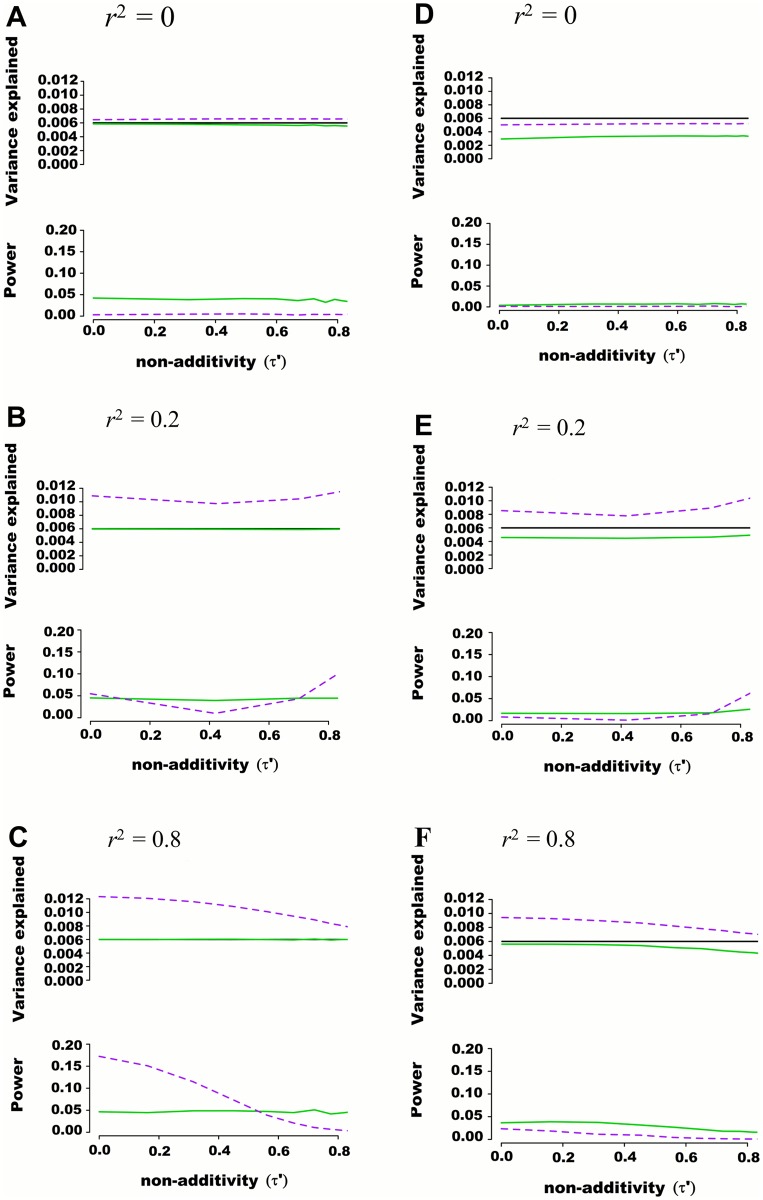
Estimated variance explained and power as a function of non-additivity measure τ*´* in regions of 100 SNPs simulated from 1000 genome data. Regions of 100 SNPs were simulated from phased 1000 Genomes data in 5,000 individuals, excluding SNPs with minor allele frequency higher than 0.01 and further pruning SNPs such that maximal pairwise linkage disequilibrium was *r* ^2^ = 0.80. Assuming two SNPs defining 4 haplotypes that are truly associated with a quantitative trait, the additive and variance component models were tested for their abilities to capture genetic variance and statistical power. The proportion of variance explained by haplotypes was fixed at 0.006 while haplotype effects varied such that the non-additivity parameter (τ*´*) ranged from 0 to 0.8. Pairwise *r* ^2^ between the 2 causal SNPs and the 98 nuisance SNPs varied from 0 to 0.25. Each scenario was simulated 10,000 times, and mean variance explained and power calculated. The frequency of haplotypes was fixed such that pairwise linkage disequilibrium between the two truly associated SNPs was either *r* ^*2*^ = 0 (A and D), *r* ^*2*^ = 0.2 (B and E) or *r* ^*2*^ = 0.8 (C and F). In figures (A), (B) and (C) the two causal SNPs were assumed to be directly genotyped along with the 98 nuisance SNPs. In figures (D), (E) and (F) the two causal SNPs were masked and only the 98 nuisance SNPs tested for association. The black line represents variance explained by the underlying haplotype model while the additive model is represented in green, and the variance-component model in dashed purple. The upper panel of each figure illustrates the estimated proportion of phenotypic variance explained by joint association as a function of non-additivity τ*´*. The lower panel illustrates the power to detect such joint association at a *p*-value threshold of 0.0001.

We also tested the ability of additive and variance component models to estimate genetic variance explained when large regions are considered. Using phased 1,000 Genomes data [16], we simulated windows of 100 SNPs, again fixing genetic variance explained at 0.006, assuming only two SNPs are truly associated with a quantitative trait, and varying non-additivity. 1,000 Genomes haplotypes were chosen from European Caucasian populations at a randomly chosen region, excluding SNPs with minor allele frequency lower than 0.01 and further pruning SNPs such that maximal pairwise linkage disequilibrium was *r* ^2^ = 0.80. Pairwise *r* ^2^ between the 2 causal SNPs and the 98 nuisance SNPs varied from 0 to 0.25. As illustrated with representative examples in Fig 2, consistent results were obtained as compared to previous scenarios including fewer SNPs, with the additive model more accurately estimating variance explained than the variance component model. This observation was also true when the 2 causal SNPs were masked, leaving only the 98 nuisance SNPs for association testing.

### Regional Association Involving Rare Genetic Variants

We evaluated the performance of regional association models to capture the phenotypic variance explained by an untyped rare SNP (MAF ≤ 0.01) when only common SNPs are directly genotyped. In these simulations, we assumed that a single rare SNP had an effect on the quantitative trait and that the proportion of variance explained was 0.0025, 0.005, 0.01 and 0.02. Two common SNPs in perfect linkage equilibrium were simulated such that together they defined a tagging haplotype with *D´* = 1 and *r* ^2^ varying from 0.24 to 1 with the rare functional SNP (*r* ^2^ between individual common SNPs and rare SNP varied from 0.03 to 0.05). We then proceeded to calculate the genetic variance captured by each association model using either only the unphased genotypes at the 2 common SNPs or further adding 3 nuisance common SNPs for a total of 5 SNPs.

The haplotype probability and genotypic model had superior power compared to the additive and variance component models. For instance, when the rare functional variant explained 0.01 of phenotypic variance and *r* ^2^ with the tagging haplotype was 1, the haplotype probability model estimated the genetic variance at 7.8x10^-3^, whereas the additive model estimated it at 8.0x10^-4^ (S1 and S2 Tables). In fact, neither the additive, interaction nor variance component model captured a significant proportion of genetic variance. All three models were underpowered to detect such an association. However, results differed if the rare functional SNP were directly genotyped and under this latter scenario, the additive, interaction and variance component models showed superior performance as compared to the haplotype and genotypic models (S3 Table).

### Large Region Joint Association with Height in HRS

We explored the contribution of large region joint associations to phenotypic variance using height. First, we individually tested each SNP for association with height in HRS, adjusting for age and sex (herein referred as a univariate analysis). As expected, given the relatively modest sample size, association *p*-values did not depart from the uniform distribution (S2A Fig). Nonetheless, when analyzing the 180 known [17] height SNPs or their best HRS proxies separately, an excess of significant *p*-values was observed (S2B Fig) although no single SNP reached genome-wide significance (*p*-value range: 9.1x10^-5^–0.99).

**Fig 3:**
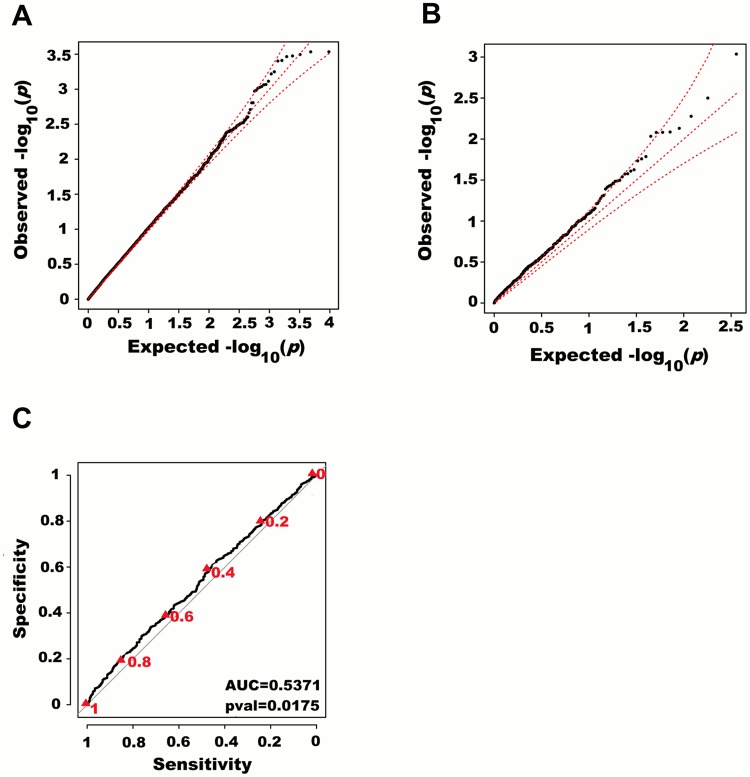
Large region joint association with height in HRS. First adjusting height for age, sex and 180 known loci, we tested for large region joint association using the previously defined additive model, setting window size at 100 SNPs with steps of 50 SNPs for a total of 9,648 windows. The quantile-quantile plot of joint association *p*-values for all windows is illustrated in (A), with 95% confidence interval. Windows encompassing each one of the 180 known loci (only) are presented in (B). Considering windows encompassing one of the 180 known height loci as true positives and all other windows as true negatives, a receiver-operating curve was constructed based on window *p*-values (C). Numbers in red represent specific window *p*-value thresholds.

We next adjusted height for all 180 known height SNPs, thus removing their main effects. We then tested for large region joint association using the previously defined additive model, setting window size at 100 SNPs with a step of 50 SNPs. A total of 9,648 windows were tested with an average size of 284.2 Kb. There was no discernable departure from the null distribution when considering all window *p*-values (Fig 3A). However, when analyzing windows encompassing known height loci separately, an excess of significant window *p*-values was observed even though all known height associations had been adjusted for (Fig 3B). Considering windows encompassing known height loci as true positives and all other windows as true negatives, the area under the receiver operating characteristic (ROC) curve for window *p*-values was 0.537 corresponding to a non-parametric *p*-value of 0.018 (Fig 3C).

**Fig 4:**
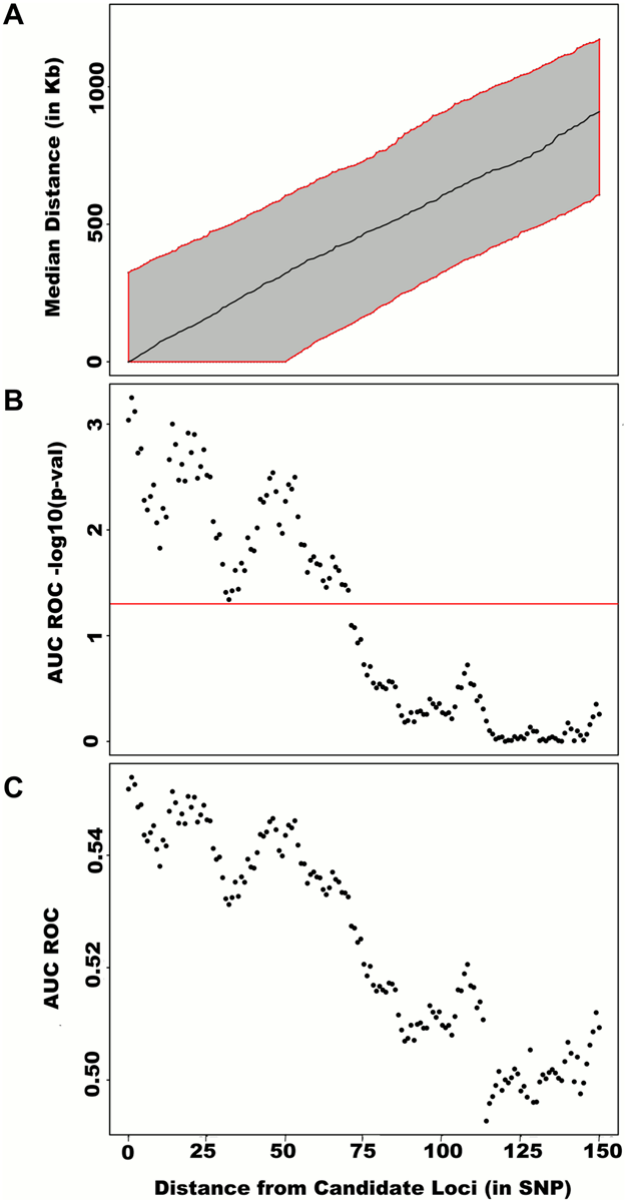
Large region joint association at known height loci. Adjusting height for age, sex and 180 known loci, we tested for large region joint association using the previously defined additive model, setting window size at 100 SNPs with steps of 1 SNP. Windows were initially centered on known height loci and distance (x-axis) was defined as the number of SNPs between the center of a window and a known height SNP. Genomic distance (in Kb) covered by windows is illustrated in (A), with red lines representing the median minimum and maximum distances between window boundaries and known height loci. Median distance between window center and known height loci is shown as the black line. In (B),—log10 *p*-value for area under the receiver operating characteristic curve is illustrated, where windows at each given distance from known height loci are compared to all 9,648 windows (the red line represents *p* = 0.05). In (C), corresponding area under the receiver operating characteristic curve is illustrated.

To assess how far away from known height loci regional associations can be detected, we centered windows on the known height SNPs and slid them away with steps of one SNP. Windows up to 71 SNPs away from the candidate SNP had a significant area under the ROC (*p* < 0.05) when compared to all other windows (Fig 4), corresponding to a median distance between window center and known height SNP of 433.0 Kb and median minimal distance between window boundary and known height SNP of 132.2 Kb. As sensitivity analyses, we varied window size (50, 75 or 100 SNPs) using height adjusted for age and sex only (S3 Fig), or additionally removing known height SNPs and their proxies (S4 Fig) instead of adjusting for known associations. Consistent results were obtained, with median minimal distance between significant windows boundary and known height SNPs larger than 100 Kb in all scenarios.

**Fig 5:**
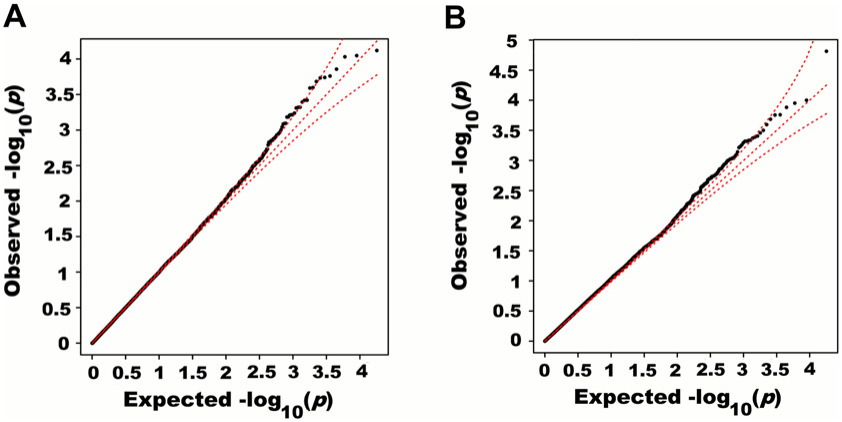
Quantile-quantile plots of association *p*-values of SNPs encompassed by windows centered on known height loci. Height was adjusted for age, sex and 180 known loci. A quantile-quantile plot of univariate association *p*-values of all SNPs encompassed by windows centered on known height loci is illustrated in (A), with 95% confidence interval. Corresponding *p*-values for the same set of SNPs but from the additive multivariate models are shown in (B).

Univariate association *p*-values from SNPs encompassed by windows centered on known height loci deviated from the uniform distribution only modestly when adjusting height for the 180 known associations (*p* = 0.0187; Fig 5A). Accordingly, no individual SNP was significant after correction for the 18,000 SNPs tested (i.e. *p*<0.05 / 18,000 = 2.8x10^-6^; lowest *p*-value = 7.6x10^-5^). However, when the corresponding SNP *p*-values were taken from regional analyses using additive multivariate models, a more pronounced excess of significant associations was observed (*p* = 0.0012; Fig 5B). In fact, the phenotypic variance explained by regional associations at the 180 known height loci was estimated at 0.172 (95% CI 0.063–0.279; *p*<0.001) through a comparison of total variance explained using real and permuted phenotype data (1,000 permutations) and assuming an additive contribution of each locus (Table 1; see Methods for details). As height was adjusted for known associations, this estimate does not include the phenotypic variance explained by these associations, which was 0.129 in HRS.

**Table 1:** Variance explained by large region joint associations in HRS.

Trait	Variance Explained by Candidate SNPs^1^	Variance explained by Candidate Windows (unadjusted^2^)		Variance explained by Candidate Windows (adjusted^3^)	
		Variance Explained (95%CI)	*p*-value^4^	Variance Explained (95%CI)	*p*-value^4^
BMI	0.0146	0.0123	0.28	-0.0019	0.52
		(-0.0259–0.0522)		(-0.0477–0.0384)	

CRP	0.0334	0.0615	<0.001	0.0376	0.01
		(0.0306–0.0901)		(0.0053–0.0663)	

HDL-C	0.0459	0.0307	0.24	-0.0150	0.64
		(-0.0544–0.1100)		(-0.0966–0.0671)	

Height	0.1292	0.3095	<0.001	0.1716	<0.001
		(0.2004–0.4070)		(0.0632–0.274)	

^1^ 28 SNPs from [18] were tested for BMI, 16 SNPs from [20] were tested for CRP, 67 SNPs from [19] were tested for HDLc, and 180 SNPs from [17] were tested for height.

^2^ In the unadjusted analysis, traits were not adjusted for candidate SNPs.

^3^ In the adjusted analysis, traits were first adjusted for candidate SNPs associations.

^4^ *P*-values were obtained from 1,000 permutations.

Since known height loci showed an excess of significant regional and univariate SNP associations, we sought to determine whether regional associations could help identify known height loci. To do so, we repeated the large region joint association analysis without adjusting for known height loci (S5 Fig). Area under the receiver operating characteristic (ROC) curve for height loci window *p*-values was 0.5901 (*p =* 8x10^-9^) as compared to all other windows. Indeed, the third most significant (*p* = 4.6x10^-4^) window encompassed the known height SNP rs974801. The most significant SNP (rs9992793) within this latter window had a univariate *p*-value of 0.0042 while rs974801 had a *p*-value of 0.029. Overall, 10% of the windows with *p* <0.01 contained a known height locus (10 out of 99 windows), corresponding to an enrichment odds ratio (*OR*) of 2.99 (95% CI 1.54–5.81; *p* = 0.001) as compared to windows with *p*>0.01. In comparison, 8.2% of the windows with *p*<0.01 contained a known height locus (8 out of 97 windows) when height was adjusted for age, sex and known height associations, corresponding to an enrichment *OR* of 2.38 (95% CI 1.14–4.95; *p* = 0.019) as compared to windows with *p* >0.01.

### Body Mass Index, High-Density Lipoprotein Cholesterol and C-reactive Protein Phenotypic Variance Explained by Large Region Joint Associations in HRS

Finally, we sought to determine whether observations made with height could be translated to other traits. We thus tested Body Mass Index (BMI) [18], High-Density Lipoprotein cholesterol (HDLc) [19] and C-reactive Protein (CRP) [20] for total variance explained by large region joint associations (Table 1). Centering windows (of size 100 SNPs) on known associations, we calculated variance explained by regional associations, with and without adjustment for known associations. While no additional variance explained was observed for BMI and HDLc, the proportion of variance explained by regional associations was estimated at 0.038 (95% CI 0.005–0.066; *p* = 0.01) for CRP after adjustment for known associations. The proportion of variance explained before adjustment was 0.062 (95% CI 0.031–0.090; *p*<0.001), which is consistent with the fraction of variance explained by known CRP SNPs in HRS of 0.033.

## Discussion

The “missing heritability” problem is one of the most pressing issues in human genetics. It is widely assumed that a large number of individually weak associations collectively explain a substantial fraction of complex trait heritability. In this report, we systematically evaluated the ability of commonly used statistical genetic models to capture large region joint associations. Our results showed that additive multivariate models have the best combination of robustness to linkage disequilibrium structure and non-additive effects while retaining adequate power. Using height data from the HRS, we then demonstrated both the presence and importance of large region joint associations using known height loci as positive controls.

Detection of regional associations in HRS is remarkable since this dataset was underpowered to identify height loci in univariate analyses, as evident from the lack of genome-wide significant results. Nonetheless, we detected large region joint associations up to 433.0 Kb away from known loci, a distance consistent with long-range *cis* regulation of gene expression. Regional associations were not the result of one or a few very significant univariate associations within tested windows; an observation supported by the modest deviation of SNP *p*-values from the uniform distribution (*p* = 0.02). This was to be expected since height was adjusted for all 180 known associations. Interestingly, a stronger (*p* = 0.001) enrichment in lower than expected *p*-values was seen when using SNP *p*-values from the multivariate additive model instead of the univariate model, even though no single SNP stood out. Taken together, these observations point to the aggregation of weak associations as the basis for joint associations, possibly combined with SNP-SNP interactions. In any case, the collective effect of these weak associations was substantial and they explained 0.17 of phenotypic variance, which compares favorably to the 0.13 explained by the 180 known height associations. Our data thus provide a further rationale for fine mapping and functional characterization of known loci. These results also suggest that regional associations could be useful to identify functional loci. Indeed, large region joint associations with known height loci were detected in HRS despite sample size being inadequate for detection of univariate associations.

Several features distinguish our approach from other methods for regional association testing. The test we propose is robust to LD although high levels of collinearity should be avoided through initial pruning of redundant SNPs (defined as *r* ^2^>0.8). This is contrast to other approaches where only SNPs in linkage equilibrium are kept (*r* ^2^<0.1–0.25) [13,21]. We also showed the equivalence between additive and variance component models when SNPs are in linkage equilibrium. This observation has significant theoretical and practical implications since additive models are computationally tractable and closed form solutions can be derived. Furthermore, estimates of genetic variance explained can be biased when using variance component analysis in the presence of LD [22], although strategies to adjust for LD have been proposed [23,24]. This is especially important in the context of regional association where strong LD is expected. Indeed, our approach sits in between popular variance component [25] and single marker approaches, combining the ability of variance component to capture overall variance explained yet providing association results for individual SNPs. In addition, our approach can cover extended regions as the degrees of freedom increase linearly with the number of SNPs in contrast to an exponential increase for genotypic and haplotype probability models, such as the one proposed by Tregouët [12]. Consequently, it is not necessary to first filter SNPs based on univariate association *p*-values [13] or condition on significant associations [7,26], an important feature as univariate SNP *p*-values followed a uniform distribution in HRS even though regional associations were present.

A few limitations are worth mentioning. First, despite demonstrating the presence of large region joint associations, additional studies will be needed to identify specific variants contributing to these associations. We propose using backward selection because variants with no or very marginal evidence of association are unlikely to contribute to regional association. However, much larger sample size will be needed, especially to assess the role of gene-gene interactions. Second, regional associations might not apply to all traits and genetic architectures might vary. Although our results support the presence of large region joint associations for height and CRP, no such association was observed for BMI and HDLc, pointing to differences in genetic architecture. Third, while variance explained by large region joint association can be estimated in empirical data using permutations, further work is needed to derive closed form solutions that are robust to linkage disequilibrium and deviation from normality. Fourth, variance explained by untyped rare variants is not well captured by our approach.

In this report, we systematically evaluated statistical methods for their ability to detect large region joint association and determined that additive models, despite their simplicity, had the most favorable profile. We then confirmed the existence of regional associations with height extending up to 433.0 Kb from known loci. Regional associations at known height loci explained 0.17 of phenotypic variance; a substantial fraction given known associations explained 0.13 in the same dataset. These results are significant as they may lead to the identification of weak associations underlying the polygenic nature of complex traits. Indeed, large region joint associations could be used to more readily identify functional regions, or conversely to further our understanding of known association loci.

## Materials and Methods

### Health Retirement Study

We conducted large region joint association analysis for height using genome-wide data from the publicly available Health Retirement Study (HRS; dbGaP Study Accession: phs000428.v1.p1). HRS quality control criteria were used for filtering of both genotype and phenotype data, namely: (1) SNPs and individuals with missingness higher than 2% were excluded, (2) related individuals were excluded, (3) only participants with self-reported European ancestry genetically confirmed by principal component analysis were included, (4) SNPs with Hardy-Weinberg equilibrium *p*<1x10^-6^ were excluded, (5) individuals for whom the reported sex does not match their genetic sex were excluded. After further pruning SNPs for LD using PLINK v.1.07 [27] with window size = 100 SNPs, step size = 50 SNPs and *r* ^2^ = 0.80, the final dataset included 3,740 European participants genotyped for 484,089 SNPs. Height was log2 transformed and adjusted for age and sex in all analyses. To mitigate the effect of outliers, we performed winsorization on log-transformed height, removing values outside the 1^st^ and 99^th^ percentile range. HRS was not part of the Genetic Investigation of Anthropometric Traits (GIANT) meta-analysis of height [15,17]. Plasma C-reactive Protein (CRP) and High Density Lipoprotein cholesterol (HDLc) were measured using standard methods in HRS. CRP, HDLc and BMI were transformed using a similar procedure as for height (including log2 transformation and winsorization) before association testing.

### Genetic Association Models

#### Additive and interaction effect models

The simple additive model follows the definition ***X*** = ***G***, where ***G*** is the previously defined genotype matrix. In addition, we construct **Ω** to be an *n* by m2 matrix representing all m2 pairwise interactions between *m* SNPs such that interactions are the product of genotypes. A more general model with interactions is given by ***X*** = $\tilde{G}$, where G~n×m+ m2 = [G Ω]. The matrix **Ω** is set to null if absence of interaction is assumed, whereas a non-null **Ω** will test for both genetic main effects and SNP-SNP interactions.

#### Genotypic models

Let $X=G_{n\;\times \;s}$ be the matrix whose *n* rows indicate which one of the *s = 3* ^*m*^ possible genotypes each of the *n* individual carries. For example, when two SNPs are considered, G will be an *n* by 9 matrix with each column representing all nine possible genotypes (i.e. 0 0, 0 1, 0 2, 1 0, etc.). $G_{i1}$ is coded as 1 if the *i* ^th^ individual has the 1^st^ specified genotype (e.g. “0 0”) and 0 otherwise.

#### Haplotype probability model

Since haplotypes are generally not experimentally observed and haplotype phasing can be ambiguous, a probabilistic approach is required to test association with haplotypes when using empirical data. Let *X* ***=*** *M*_**n×k**_ be the matrix whose *n* rows represent the expected number of *k* possible haplotypes for each of the *n* individuals. In other words, when haplotype phasing is non-ambiguous, each row entry will be 0, 1 or 2 and the row sum will be 2. On the other hand, when haplotype phasing is ambiguous, row entries will take values between 0 and 2, corresponding to the expected number of each possible haplotype such that the row sum will be 2.

#### Variance component model

The variance explained by SNPs can be estimated using variance component (VC) models [2]. The variance-covariance matrix of ***Y*** can be expressed as

$$Var\left(Y\right)\;=\;\frac{\Gamma \sigma_{g}^{2}}{m}+I\sigma_{e}^{2},$$

with ***I*** the identity matrix, $\sigma_{e}^{2}$ the residual variance, $\sigma_{g}^{2}$ the variance of total additive genetic effects, and ***Γ* = *ZZ′***, an *n* by *n* symmetric matrix whose entries ***Γ***_*i*,*j*_ represent the genetic similarity between individuals *i* and *j*. Each entry of the matrix ***Z*** is the normalized genotypes

zil = -2 ξl2 ξl(1- ξl) ( gil = 0)  1-2 ξl2 ξl(1- ξl) ( gil = 1) 2-2 ξl2 ξl(1- ξl) ( gil = 2)

such that E(z_il_) = 0 and Var(z_il_) = 1, where $\xi_{l}$ is the allele frequency of the *l* ^th^ SNP. The variance component model can be alternatively written [2] as $\vec{\Delta }Y_{\left(n^{2}\times 1\right)}^{2}\;=\;v_{0}+v_{1}{\vec{\Gamma }}_{\left(n^{2}\times 1\right)}+\varepsilon$, where $\Delta y_{ij}^{2}\;=\;{\left(y_{i}-y_{j}\right)}^{2}$ is the squared pairwise difference of the trait *for* all possible pairs of individuals *i* and *j*, and ${\vec{\Gamma }}_{\left(n^{2}\times 1\right)}$ is a vectorized form of the ***Γ*** matrix by row. The regression coefficients υ_0_ and υ_1_ can be estimated from observed data and it has been shown [2] that $v_{1}\;=\;-2\sigma_{g}^{2}$.

### Equivalence between Additive Model and Variance Component Model

The additive model can be shown to be equivalent to the variance component model when all SNPs are in linkage equilibrium (i.e. the variance-covariance matrix of ***Z*** is the identity matrix). In this case, the genetic variance explained by the model **Y** = **Zβ + ε** is given by:

$${\hat{Y}}^{'}\hat{Y}\;=\;{\left[Z\hat{\beta }\right]}^{'}\left[Z\hat{\beta }\right]\;=\;{\left[Z{\left(Z^{'}Z\right)}^{-1}Z^{'}Y\right]}^{'}\left[Z{\left(Z^{'}Z\right)}^{-1}Z^{'}Y\right]$$

$$=\;Y^{'}Z{\left(Z^{'}Z\right)}^{-1}Z^{'}Y\;=\;Y^{'}ZZ^{'}Y\;=\;Y^{'}\Gamma Y$$

$$=\;\sum_{i\;=\;1}^{n}\sum_{j\;=\;1}^{n}y_{i}y_{j}\;\Gamma_{i,j}$$

$$=\;\frac{\sigma_{g}^{2}}{m}\sum_{i\;=\;1}^{n^{2}}{\vec{\Gamma }}_{i}^{2}$$

The latter derivation assumes all individuals are unrelated (as done throughout the manuscript). Should participants be related, the variance component model would remain appropriate while the additive model would not.

### Variance Explained by Genetic Variants and Statistical Power

Genetic association models can be used to estimate the phenotypic variance explained by genetic variants, commonly expressed as the ratio of the genetic variance and total variance, and herein denoted as *R* ^2^. As previously defined, the true underlying genetic model is expressed as **Y** = **Dβ + ε,** where ***D*** is the matrix of true (unobserved) haplotypes, ***β*** is the *k* × 1vector of haplotype effects, π_i_ the frequency of the *i*th haplotype, and ε the standard normally distributed random error. The total variance is given by: VarY = VarDβ+Varε = σH2+1 = ∑i = 1kβi2(2πi(1-πi))-4∑i = 1 j>ikβiβjπiπj+1. Genetic variance $\sigma_{X}^{2}$ captured by association testing can be calculated for each specific association model used, such that $R^{2}\;=\;\frac{\sigma_{X}^{2}}{\sigma_{H}^{2}+1}$. Power estimates for additive, interaction, genotypic and haplotype probability models can be obtained using the non-central *F*-distribution with a non-centrality parameter $\frac{nR^{2}}{\left(1-R^{2}\right)}$ [28], where *n* is the number of individuals. However, the variance component model has a quadratic form *Q* = ***Y*′ΓY** (i.e. a linear combination of chi-squared random variables) and the non-central *F*-distribution is not appropriate. In light of Duchesne and Lafaye De Micheaux [29], *Q* can be expressed as a non-central chi-squared random variable with *m* degrees of freedom (with *m* corresponding to the number of SNPs). Several approximations and exact methods have been suggested for weighted sum of chi-squared random variables and among these, Davies’ exact method [30] appears to work well in empirical settings [25].

### Phenotypic Variance Explained by Regional Associations

Total variance explained by regional associations was estimated using a permutation procedure. Briefly, variance explained by each window was first estimated on real, non-permuted, phenotypes. Phenotypes were then permuted 1,000 times (preserving the linkage disequilibrium structure of SNPs) and variance explained estimated on permuted phenotypes. Adjusted variance explained by each window was then defined as the difference between variance explained using real, non-permuted, phenotypes and the mean variance explained by the corresponding window when testing permuted phenotypes. Total variance explained was then calculated as the sum of adjusted variance explained by each window. That is, each locus (i.e. window) was assumed to additively contribute to total variance explained, regardless of other loci. This permutation procedure was used to ensure neither linkage disequilibrium nor deviation of the phenotype from normality would inflate results. This is relevant since each window individually contributes only modestly to variance explained. Furthermore, the large number of SNPs included across all candidate windows precludes testing all SNPs at once, thus motivating the use of variance explained per window.

## Supporting Information

S1 Fig Haplotype effect as a function of non-additivity measure τ′. A quantitative trait was assumed to be genetically determined according to the underlying (unobserved) haplotype model ***Y*** *=* ***Dβ + ε***, where 2 SNPs define 4 possible haplotypes. The proportion of variance explained by haplotypes was fixed at 0.006 while haplotype effects varied such that the non-additivity parameter (τ′) ranged from 0 to 1. In (A), the frequency of the 4 haplotypes was fixed such that pairwise linkage disequilibrium between SNPs was *r* ^*2*^ = 0, corresponding to haplotype frequencies of π_1_ = 0.10 (red), π_2_ = 0.30 (blue), π_3_ = 0.15 (green) and π_4_ = 0.45 (black). In (B), frequencies were fixed such that *r* ^*2*^ = 0.2, corresponding to haplotype frequencies of π_1_ = 0.42 (red), π_2_ = 0.18 (blue), π_3_ = 0.10 (green) and π_4_ = 0.30 (black).
(TIF)

S2 Fig Quantile-quantile plots of univariate SNP association *p*-values with height in HRS, adjusting for age and sex. In (A), *p*-values for all 484,089 SNPs are illustrated. In (B), only *p*-values for the 180 known height SNPs or their best HRS proxies are shown.
(TIF)

S3 Fig Large region joint association at known height loci, adjusting for age and sex only. Adjusting height for age and sex only, we tested for large region joint association using the previously defined additive model, setting window size at 50 (A), 75 (B) or 100 (C) SNPs with steps of 1 SNP. Windows were initially centered on known height loci and distance (x-axis) was defined as the number of SNPs between the center of a window and a known height SNP. Genomic distance (in Kb) covered by windows is illustrated in upper panels, with red lines representing the median minimum and maximum distances between window boundaries and known height loci. Median distance between window center and known height loci is shown as the black line. In middle panels,—log10 *p*-value for area under the receiver operating characteristic curve is illustrated, where windows at each given distance from known height loci are compared to all 9,648 windows (the red line represents *p* = 0.05). In lower panels, corresponding area under the receiver operating characteristic curve is illustrated.
(PPTX)

S4 Fig Large region joint association at known height loci, adjusting for age and sex only but removing known height loci. Adjusting height for age and sex only but removing all SNPs with *r* ^*2*^ >0.1 with a known height SNP (within 500 Kb), we tested for large region joint association using the previously defined additive model. Window size was set at 50 (A), 75 (B) or 100 (C) SNPs, with steps of 1 SNP. Windows were initially centered on known height loci and distance (x-axis) was defined as the number of SNPs between the center of a window and a known height SNP. Genomic distance (in Kb) covered by windows is illustrated in upper panels, with red lines representing the median minimum and maximum distances between window boundaries and known height loci. Median distance between window center and known height loci is shown as the black line. In middle panels,—log10 *p*-value for area under the receiver operating characteristic curve is illustrated, where windows at each given distance from known height loci are compared to all 9,648 windows (the red line represents *p* = 0.05). In lower panels, corresponding area under the receiver operating characteristic curve is illustrated.
(TIF)

S5 Fig Large region joint association with height in HRS, adjusting for age and sex only. Adjusting height for age and sex only, we tested for large region joint association using the previously defined additive model. Window size was set at 50 SNPs with steps of 25 (A, D and G), 100 with steps of 75 (B, E and H) or 150 with steps of 75 (C, F and I). Quantile-quantile plots of joint association *p*-values for all tested windows are illustrated in A, B and C. Quantile-quantile plots for windows encompassing each one of the 180 known loci (only) are presented in D, E and F. Considering windows encompassing one of the 180 known height loci as true positives and all other windows as true negatives, receiver operating characteristic curves were constructed based on window *p*-values (G, H and I). Numbers in red represent specific window *p*-value thresholds.
(PPTX)

S1 Table Power and estimated proportion of variance explained by joint association of two common SNPs tagging a single untyped rare functional genetic variant. (DOCX)

S2 Table Power and estimated proportion of variance explained by joint association of five common SNPs when two of the five common SNPs tag a single untyped rare functional genetic variant. (DOCX)

S3 Table Power and estimated proportion of variance explained by joint association of five common SNPs when two of the five common SNPs tag a single typed rare functional genetic variant. (DOCX)
