# Supplementary figures and images for: Contribution of Large Region Joint Associations to Complex Traits Genetics
Source: PLoS Genet. 2015 Apr 9;11(4):e1005103. doi: 10.1371/journal.pgen.1005103 (PMC4391841; doi:10.1371/journal.pgen.1005103)

## Slide 1
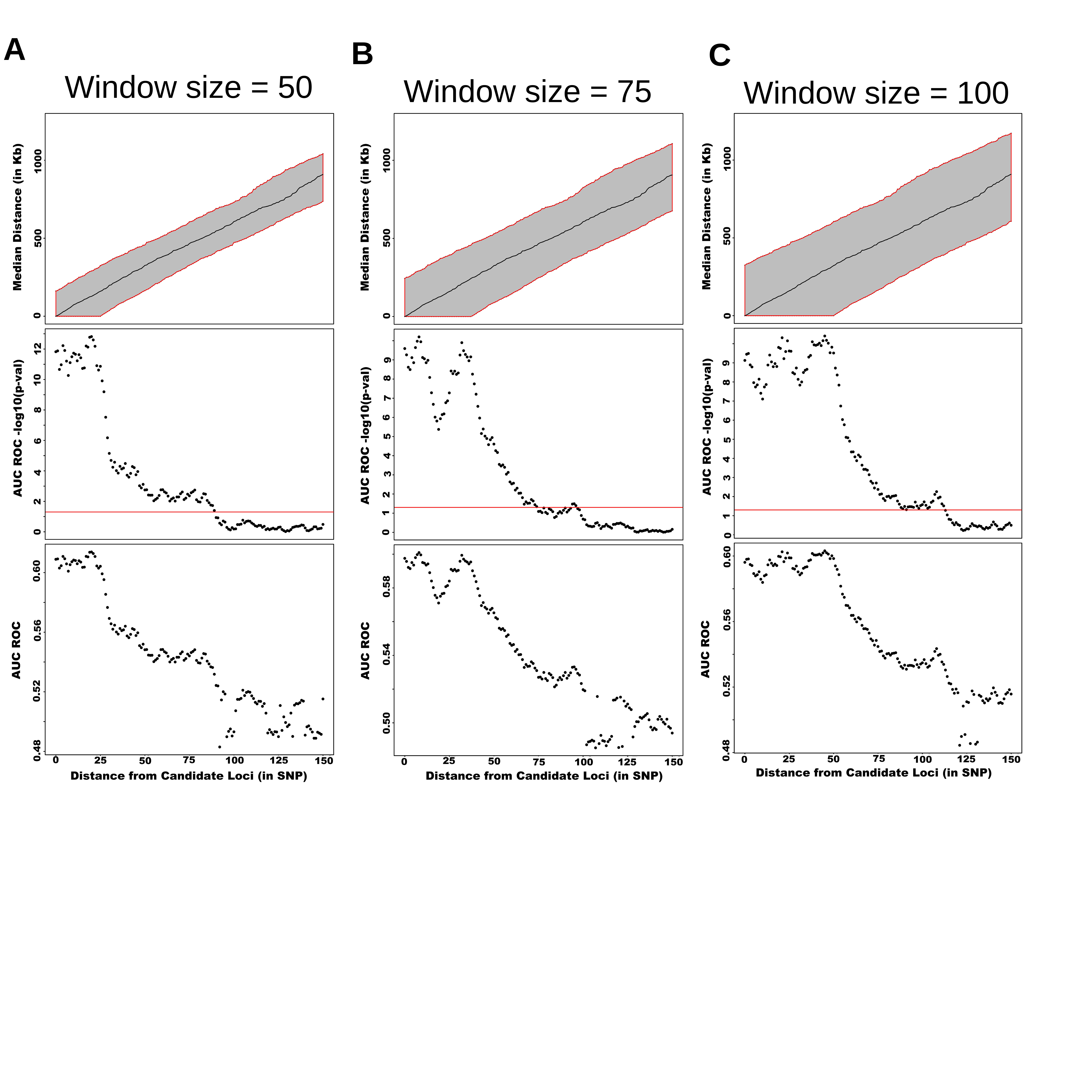

A
 Window size = 50 SNPs
B
 Window size = 75 SNPs
C
 Window size = 100 SNPs

Supplement: S3 Fig — Adjusting height for age and sex only, we tested for large region joint association using the previously defined additive model, setting window size at 50 (A), 75 (B) or 100 (C) SNPs with steps of 1 SNP. Windows were initially centered on known height loci and distance (x-axis) was defined as the number of SNPs between the center of a window and a known height SNP. Genomic distance (in Kb) covered by windows is illustrated in upper panels, with red lines representing the median minimum and maximum distances between window boundaries and known height loci. Median distance between window center and known height loci is shown as the black line. In middle panels,—log10 p-value for area under the receiver operating characteristic curve is illustrated, where windows at each given distance from known height loci are compared to all 9,648 windows (the red line represents p = 0.05). In lower panels, corresponding area under the receiver operating characteristic curve is illustrated. (PPTX) [file pgen.1005103.s003.pptx]

## Slide 1
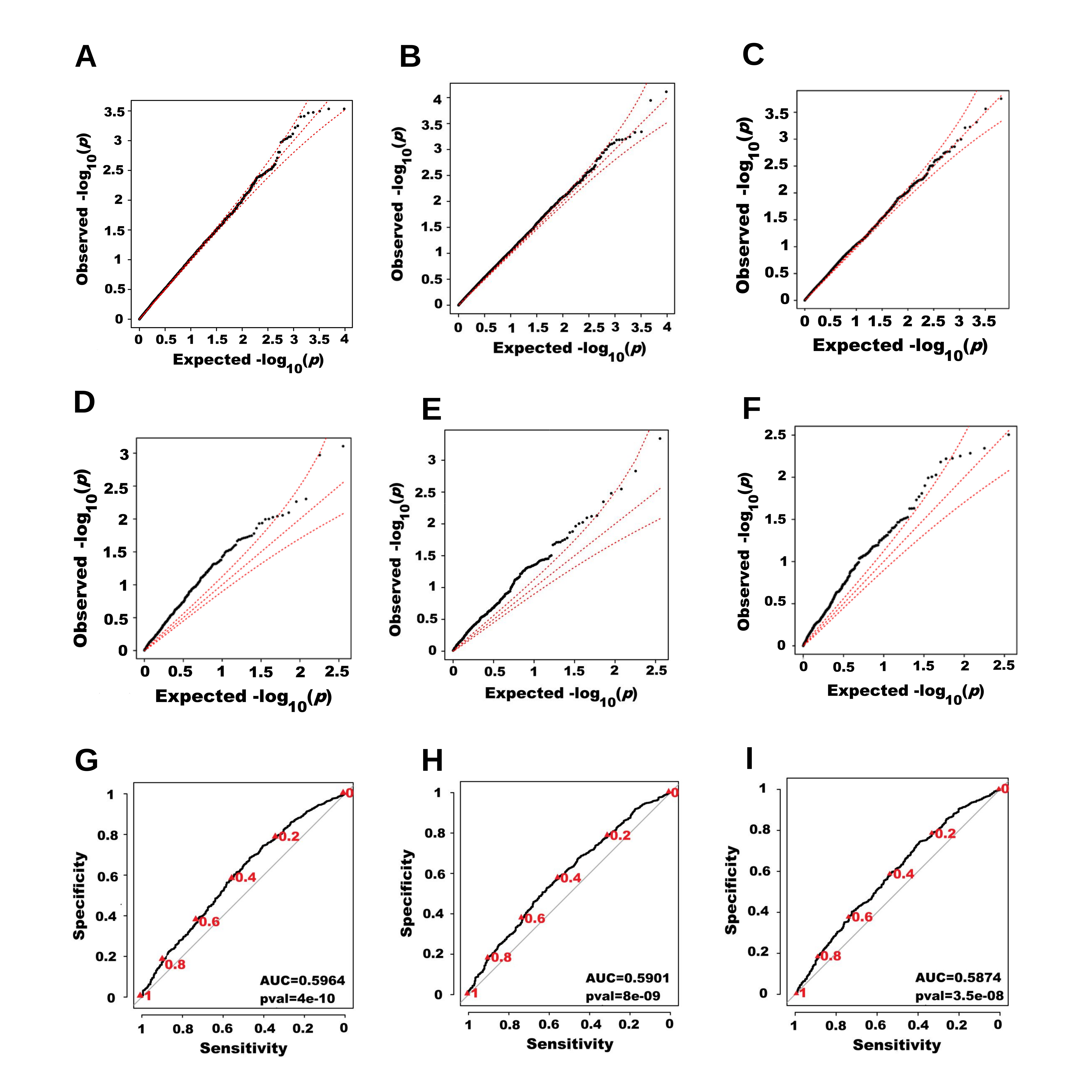

C
A
B
D
F
E
I
G
H

Supplement: S5 Fig — Adjusting height for age and sex only, we tested for large region joint association using the previously defined additive model. Window size was set at 50 SNPs with steps of 25 (A, D and G), 100 with steps of 75 (B, E and H) or 150 with steps of 75 (C, F and I). Quantile-quantile plots of joint association p-values for all tested windows are illustrated in A, B and C. Quantile-quantile plots for windows encompassing each one of the 180 known loci (only) are presented in D, E and F. Considering windows encompassing one of the 180 known height loci as true positives and all other windows as true negatives, receiver operating characteristic curves were constructed based on window p-values (G, H and I). Numbers in red represent specific window p-value thresholds. (PPTX) [file pgen.1005103.s005.pptx]
