# Supplementary material for: Contribution of Large Region Joint Associations to Complex Traits Genetics
Source: PLoS Genet. 2015 Apr 9;11(4):e1005103. doi: 10.1371/journal.pgen.1005103 (PMC4391841; doi:10.1371/journal.pgen.1005103)
Supplement: S1 Table — (DOCX) [file pgen.1005103.s006.docx]

**Table S1**: Power and estimated proportion of variance explained by joint association of two common SNPs tagging a single untyped rare functional genetic variant.

|  | | | Power | | | | | Variance Explained | | | | |
| --- | --- | --- | --- | --- | --- | --- | --- | --- | --- | --- | --- | --- |
| Effect  Size | Frequency of tagging haplotype  $(\pi_{\mathrm{tag}})$ | Effect size under haplotype model | Haplotype  Probability  Model | Additive  Model | Interaction  Model | Genotypic  Model | Variance  Component  Model | Haplotype  Probability  Model | Additive  Model | Interaction  Model | Genotypic  Model | Variance  Component  Model |
| 0.0025 | 0.01 | 0.0025 | 0.066241 | 0.000623 | 0.001895 | 0.027256 | 6.05E-05 | 0.001945 | 0.0002 | 0.000446 | 0.001945 | 5.87E-05 |
|  | 0.02 | 0.001239 | 0.009401 | 0.000554 | 0.001316 | 0.003582 | 6.03E-05 | 0.000977 | 0.000185 | 0.000374 | 0.000977 | 5.70E-05 |
|  | 0.04 | 0.000607 | 0.001732 | 0.000452 | 0.000767 | 0.000726 | 5.98E-05 | 0.000491 | 0.000162 | 0.000281 | 0.000491 | 5.35E-05 |
| 0.005 | 0.01 | 0.005 | 0.394694 | 0.002223 | 0.009999 | 0.223217 | 7.61E-05 | 0.003889 | 0.0004 | 0.000893 | 0.003889 | 0.000117 |
|  | 0.02 | 0.002481 | 0.067399 | 0.001909 | 0.006366 | 0.027779 | 7.55E-05 | 0.001956 | 0.000371 | 0.00075 | 0.001956 | 0.000114 |
|  | 0.04 | 0.001217 | 0.009589 | 0.001455 | 0.003179 | 0.003653 | 7.43E-05 | 0.000984 | 0.000324 | 0.000563 | 0.000984 | 0.000107 |
| 0.01 | 0.01 | 0.01 | 0.935847 | 0.011116 | 0.067324 | 0.830416 | 0.000111 | 0.007778 | 0.000799 | 0.001786 | 0.007778 | 0.000235 |
|  | 0.02 | 0.004975 | 0.401788 | 0.009288 | 0.041619 | 0.228468 | 0.000109 | 0.003923 | 0.000744 | 0.001503 | 0.003923 | 0.000229 |
|  | 0.04 | 0.002443 | 0.069325 | 0.006659 | 0.018859 | 0.028652 | 0.000107 | 0.001976 | 0.00065 | 0.00113 | 0.001976 | 0.000215 |
| 0.02 | 0.01 | 0.02 | 0.999979 | 0.069135 | 0.383643 | 0.99974 | 0.000253 | 0.015557 | 0.001599 | 0.003571 | 0.015557 | 0.000469 |
|  | 0.02 | 0.01 | 0.940985 | 0.057922 | 0.265391 | 0.840559 | 0.00024 | 0.007886 | 0.001496 | 0.003021 | 0.007886 | 0.00046 |
|  | 0.04 | 0.004923 | 0.41432 | 0.040577 | 0.130627 | 0.237857 | 0.000223 | 0.003982 | 0.00131 | 0.002278 | 0.003982 | 0.000434 |
