# Supplementary material for: Contribution of Large Region Joint Associations to Complex Traits Genetics
Source: PLoS Genet. 2015 Apr 9;11(4):e1005103. doi: 10.1371/journal.pgen.1005103 (PMC4391841; doi:10.1371/journal.pgen.1005103)
Supplement: S2 Table — (DOCX) [file pgen.1005103.s007.docx]

**Table S2**: Power and estimated proportion of variance explained by joint association of five common SNPs when two of the five common SNPs tag a single untyped rare functional genetic variant.

|  | | | Power | | | | | Variance Explained | | | | |
| --- | --- | --- | --- | --- | --- | --- | --- | --- | --- | --- | --- | --- |
| Effect  Size | Frequency of tagging haplotype  $(\pi_{\mathrm{tag}})$ | Effect size under haplotype model | Haplotype  Probability  Model | Additive  Model | Interaction  Model | Genotypic  Model | Variance  Component  Model | Haplotype  Probability  Model | Additive  Model | Interaction  Model | Genotypic  Model | Variance  Component  Model |
| 0.0025 | 0.01 | 0.0025 | 0.000427 | 0.000181 | 0.000228 | 0.000105 | 0.000112 | 0.000754 | 0.000135 | 0.000323 | 0.000754 | 0.000111 |
|  | 0.02 | 0.001239 | 0.000198 | 0.00017 | 0.00019 | 7.82E-05 | 0.000116 | 0.000452 | 0.000127 | 0.000279 | 0.000452 | 0.000108 |
|  | 0.04 | 0.000607 | 0.000121 | 0.000152 | 0.000147 | 6.60E-05 | 0.000121 | 0.000278 | 0.000113 | 0.000217 | 0.000278 | 0.000102 |
| 0.005 | 0.01 | 0.005 | 0.002061 | 0.000446 | 0.000716 | 0.000208 | 0.000216 | 0.001508 | 0.000269 | 0.000645 | 0.001508 | 0.000222 |
|  | 0.02 | 0.002481 | 0.000606 | 0.000407 | 0.000539 | 0.00012 | 0.000225 | 0.000904 | 0.000254 | 0.000558 | 0.000904 | 0.000216 |
|  | 0.04 | 0.001217 | 0.000262 | 0.000344 | 0.000351 | 8.66E-05 | 0.000238 | 0.000557 | 0.000227 | 0.000436 | 0.000557 | 0.000205 |
| 0.01 | 0.01 | 0.01 | 0.018615 | 0.001668 | 0.003911 | 0.000729 | 0.000658 | 0.003016 | 0.000538 | 0.00129 | 0.003016 | 0.000443 |
|  | 0.02 | 0.004975 | 0.003499 | 0.001475 | 0.002631 | 0.000272 | 0.000686 | 0.001813 | 0.000509 | 0.001118 | 0.001813 | 0.000434 |
|  | 0.04 | 0.002443 | 0.000964 | 0.001163 | 0.001407 | 0.000147 | 0.000716 | 0.001119 | 0.000455 | 0.000875 | 0.001119 | 0.000412 |
| 0.02 | 0.01 | 0.02 | 0.206665 | 0.009563 | 0.034518 | 0.005732 | 0.003679 | 0.006033 | 0.001076 | 0.002581 | 0.006033 | 0.000886 |
|  | 0.02 | 0.01 | 0.036459 | 0.008302 | 0.021874 | 0.001172 | 0.003727 | 0.003645 | 0.001023 | 0.002248 | 0.003645 | 0.000873 |
|  | 0.04 | 0.004923 | 0.006923 | 0.006173 | 0.009987 | 0.000395 | 0.003691 | 0.002254 | 0.000917 | 0.001763 | 0.002254 | 0.000831 |
