# Supplementary material for: Contribution of Large Region Joint Associations to Complex Traits Genetics
Source: PLoS Genet. 2015 Apr 9;11(4):e1005103. doi: 10.1371/journal.pgen.1005103 (PMC4391841; doi:10.1371/journal.pgen.1005103)
Supplement: S3 Table — (DOCX) [file pgen.1005103.s008.docx]

**Table S3**: Power and estimated proportion of variance explained by joint association of five common SNPs when two of the five common SNPs tag a single typed rare functional genetic variant.

|  | | | Power | | | | | Variance Explained | | | | |
| --- | --- | --- | --- | --- | --- | --- | --- | --- | --- | --- | --- | --- |
| Effect  Size | Frequency of tagging haplotype  $(\pi_{\mathrm{tag}})$ | Effect size under haplotype model | Haplotype  Probability  Model | Additive  Model | Interaction  Model | Genotypic  Model | Variance  Component  Model | Haplotype  Probability  Model | Additive  Model | Interaction  Model | Genotypic  Model | Variance  Component  Model |
| 0.0025 | 0.01 | 0.0025 | 0.0031 | 0.0930 | 0.0189 | 0.0001 | 0.1001 | 0.0025 | 0.0025 | 0.0025 | 0.0025 | 0.0025 |
|  | 0.02 | 0.001239 | 0.0031 | 0.0930 | 0.0189 | 0.0001 | 0.0996 | 0.0025 | 0.0025 | 0.0025 | 0.0025 | 0.0025 |
|  | 0.04 | 0.000607 | 0.0031 | 0.0930 | 0.0189 | 0.0001 | 0.0977 | 0.0025 | 0.0025 | 0.0025 | 0.0025 | 0.0025 |
| 0.005 | 0.01 | 0.005 | 0.0359 | 0.5241 | 0.1927 | 0.0005 | 0.5359 | 0.005 | 0.005 | 0.005 | 0.005 | 0.0051 |
|  | 0.02 | 0.002481 | 0.0359 | 0.5241 | 0.1927 | 0.0005 | 0.5348 | 0.005 | 0.005 | 0.005 | 0.005 | 0.0051 |
|  | 0.04 | 0.001217 | 0.0359 | 0.5241 | 0.1927 | 0.0005 | 0.5300 | 0.005 | 0.005 | 0.005 | 0.005 | 0.0050 |
| 0.01 | 0.01 | 0.01 | 0.3993 | 0.9809 | 0.8354 | 0.0038 | 0.9816 | 0.01 | 0.01 | 0.01 | 0.01 | 0.0102 |
|  | 0.02 | 0.004975 | 0.3993 | 0.9809 | 0.8354 | 0.0038 | 0.9815 | 0.01 | 0.01 | 0.01 | 0.01 | 0.0102 |
|  | 0.04 | 0.002443 | 0.3993 | 0.9809 | 0.8354 | 0.0038 | 0.9809 | 0.01 | 0.01 | 0.01 | 0.01 | 0.0101 |
| 0.02 | 0.01 | 0.02 | 0.9861 | 1 | 0.9999 | 0.0658 | 1 | 0.02 | 0.02 | 0.02 | 0.02 | 0.0204 |
|  | 0.02 | 0.01 | 0.9861 | 1 | 0.9999 | 0.0658 | 1 | 0.02 | 0.02 | 0.02 | 0.02 | 0.0204 |
|  | 0.04 | 0.004923 | 0.9861 | 1 | 0.9999 | 0.0658 | 1 | 0.02 | 0.02 | 0.02 | 0.02 | 0.0203 |
